# Conscience and Consistency

**Published:** 1883-08

**Authors:** E. P. Hussey

**Affiliations:** Buffalo, N. Y.


### CONSCIENCE AND CONSISTENCY.

The editors of the *Hahnemannian Monthly* have placed themselves in a deplorable position, one in which it is impossible for them to be either consistent or honest. It was expected they would have their fling at the Homœopathic Medical Society of Central New York on account of its protest against "that resolution," but we hardly looked for such a complete perversion of the intent of the Society's action as appears in the criticism on page 376 of the June number of the above-mentioned journal. The protest was prescribed for *just* those "weak knees" which cannot "walk upright without" (or with) "artificial support," and the "resolution" certainly demonstrates who need the prescription. As for their "own conscience" being sufficient to keep them from disobedience to the law of cure, or a reliable guide in any other matter, we are taught that conscience is chiefly a matter of education; and in this particular instance it seems to be educated in such a way as to be something of an unknown quantity, developing at one time a resolution, the known object of which is the substitution of that Will o' the Wisp, "freedom of medical opinion," for duty to a known law, and again giving us the following guiding principles of the members of the "Central" Society, and of every homœopathician: "Hahnemann's discovery is a law of nature, as immutable as truth itself. And those of us who believe in it must defend it faithfully, unitedly and *intelligently*, if we would be good stewards and honest men, and would save our school from insidious foes without and traitors within." Are the men who framed, or the Society which adopted a resolution, the tendency of which is to prostitute the results of Hahnemann's discovery to quackery, "good stewards and honest men"? And now, we are told, the author of the "resolution" says it "refers solely to the consultation question!"—a new phase of an education that will allow its possessor to pander to the low sentiment of popularity at the sacrifice of known duty. What consistency is there between that and all it implies, and the article entitled *Absolution*, in the *Hahnemannian Monthly*, June, 1883, page 372, from which we quoted above? Surely there is need of protest, sore need for those who are not afraid of being consistent, to raise their voices and the light of truth in the fog and mist that is threatening us.

E. P. Hussey, M. D.,
Buffalo, N. Y.

