# Errata

**Published:** 1883-09

**Authors:** 


Errata.—August number, page 240, eight lines from bottom, after *education* insert *of conscience.*

